# Simulations of Heart Function

**DOI:** 10.1155/2015/626378

**Published:** 2015-11-12

**Authors:** Rodrigo Weber dos Santos, Sergio Alonso, Elizabeth M. Cherry, Joakim Sundnes

**Affiliations:** ^1^Graduate Program in Computational Modeling, Federal University of Juiz de Fora, 36036-330 Juiz de Fora, MG, Brazil; ^2^Department of Applied Physics, Polytechnic University of Catalonia, Avenida Dr. Marañón 44, 08028 Barcelona, Spain; ^3^School of Mathematical Sciences, Rochester Institute of Technology, 85 Lomb Memorial Drive, Rochester, NY 14623, USA; ^4^Simula Research Laboratory, Martin Linges Vei 17, 1325 Lysaker, Norway; ^5^Department of Informatics, University of Oslo, Gaustadalléen 23B, 0373 Oslo, Norway

The heart is a robust and reliable organ that, approximately once per second, pumps the blood to the whole body. This activity involves the fine coupling of numerous components involving a large variety of physical processes and covering a wide range of scales. For instance, heart function depends on cell metabolism, electrophysiology, and mechanics; ion channels, ion concentrations, and gap junction distribution; material and electrical properties of cardiac tissue and the anatomical organization of this tissue in fibers and sheets; and material properties and organization of the heart's vascular tree and their relation to cardiac perfusion. Understanding the details of these complex systems, as well as the interactions among them, is crucial for understanding heart function in health and disease.

This special issue focuses on cardiac modeling and simulations that can contribute to improving the understanding of this multifaceted system under normal conditions and different cardiac pathologies. Multiple types of computational and mathematical models are used to describe heart function at different levels of details. For instance, relatively simple models have been employed to characterize the main properties of action potential propagation and wave dynamics in cardiac tissue, and detailed physiological models have been employed to improve our understanding of arrhythmia generation, fibrillation, and defibrillation. Coupled models of cardiac electromechanics that involve multiple scales, from intracellular to whole-organ, have been developed to describe the relation between electric signals and heart contraction. In summary, cardiac modeling has advanced over several decades to become a valuable tool for studying heart function.

The articles in this issue span a broad range of topics related to simulating heart function. On the modeling side, a detailed mathematical description of the heart's function involves the combination of different models, such as the electrophysiology of membrane potential of cardiac cells, the mechanics of the contracting tissue, and the fluid dynamics of the blood inside the chambers of the heart. Such combination requires integrating descriptions of different physical mechanisms, via multiphysics models, and the consideration of processes at different scales, from individual ion channels to the motion of the heart inside the human body, via multiscale models. Mathematical models are required for all these processes and scales. For example, in this special issue, A. Jalali et al. apply mathematical modeling to the pressure changes controlling the flow dynamics in the heart and in particular the effects in the case of a pathological size of the left ventricle. The coupling of the electrical signal with mechanics is modeled by B. M. Rocha et al. to study changes in mechanoelectric feedback due to heart failure and by P. Brocklehurst et al. to study human atrial tissue and atrial fibrillation using discrete approaches.

Several articles include findings with the potential for clinical relevance. The results presented by B. M. Rocha et al. suggest that, in both normal and heart failure conditions, the information carried by the T-wave is highly related to cardiac contraction—that is, electrocardiogram (ECG) waveforms carry not only information of cardiac electric activity but also information of cardiac contraction. However, the interpretation of ECGs taking contraction into account is not straightforward for clinicians to perform using currently available tools. Translation to clinical practice will require the use of modern models of cardiac electromechanics that consider nonlinear relations between contraction and electrical excitation of the heart.

In another ECG study, A. Loewe et al. simulated 12-lead ECGs to investigate the detection of early myocardial ischemia. To this end, several simulations were performed to represent different locations and sizes of ischemic regions in the left ventricle. With these data, the authors tested different setups and locations of ECG leads to optimize the detection of early ischemic episodes. Improvements were reported from adding a single extra electrode, and these findings suggest the need for further study of the setup of ECG leads in clinical practice.

P. Ganesan et al. focus on the use of electrograms acquired by invasive multipolar diagnostic catheters during clinical electrophysiology studies of atrial arrhythmias. P. Ganesan et al. use both simulated and clinically acquired electrograms to study how these waveforms are related to the location of the region in the atria that can be targeted for ablation, that is, a region that sustains the arrhythmia. They present an algorithm that uses electrogram information to guide the catheter toward these arrhythmia-sustaining regions, which in the case of atrial fibrillation then can be targets of ablation during the same clinical electrophysiological studies.

The characterization and study of different types of arrhythmias are the focus of a number of other papers in this special issue. For instance, one of the main explanations for the formation of reentrant waves is cardiac alternans. K. Kulkarni et al. use an alternate pacing protocol to show that the bifurcation to alternans for isolated whole rabbit hearts occurs via a smooth bifurcation rather than a border-collision mechanism. For the case of reentry in three-dimensional tissue, the dynamics of the filaments governing such reentries may determine the persistence of the arrhythmia. Filaments and waves resulting from numerical simulations are studied by P. Pathmanathan and R. A. Gray for rabbit and by S. Pravdin et al. and S. R. Kharche et al. for human hearts. P. Pathmanthan and R. A. Gray use a high-resolution biventricular anatomical model to show that fine-scale anatomical details can affect scroll-wave dynamics through behavior such as filament breakup, reattachment, anchoring, and lengthening. In another study focusing on the effects of ventricular anatomy, S. Pravdin et al. study how filament tension (positive versus negative), ventricular shape, and strength of anisotropy determine filament location. S. R. Kharche et al. show that anatomical structures can affect scroll-wave dynamics in atria; variations in tissue thickness, such as at pectinate muscle bridges, can cause breakthrough patterns and transiently anchor waves. Characterizations of wave dynamics in these ways can be useful for the study of the influence of pathological conditions on the dynamics of the waves. For example, A. Bueno-Orovio et al. study the conditions needed for phase-2 reentry to give rise to tissue-level arrhythmias in the form of spiral and scroll waves in the context of electrophysiological changes associated with Brugada syndrome.

At the mesoscopic scale, the network formed by the Purkinje fibers, which propagates the electrical signal from the atria to the ventricles, becomes relevant. Two articles address ways to model and simulate the Purkinje system. W. Ying and C. S. Henriquez propose and test a new adaptive method to improve the efficiency of simulations of electrical activity in Purkinje fibers while retaining accuracy. Because the topological features of the Purkinje network may vary significantly from one individual to another, B. R. Liu and E. M. Cherry propose a new method for generating three-dimensional Purkinje networks based on imaging data, that is, on histological data. This approach was used to create models of the combined ventricle-Purkinje system, which have the potential to help elucidate the role of Purkinje network during ventricular arrhythmias.

The continuous approach in the macroscopic limit normally used by mathematical models is clearly broken when the microscopic details of single cells are to be taken into account. P. Brocklehurst et al. use a discrete approach in modeling human atrial tissue. Another interesting question that is difficult to address via continuum models is why fibrotic regions of the heart are usually associated with ectopic beats or triggers of arrhythmia. One possible mechanism suggests that fibroblasts promote arrhythmogenesis through direct electrical interactions with cardiomyocytes via gap junctions. T. R. Brown et al. present a review on this specific topic as well as new results on how voltage-dependent gap junctions affect fibroblast-myocyte interactions.

Another suggested mechanism is that fibroblasts produce excess extracellular matrix that slows and fractionates propagation, causing zig-zag conduction paths that may act as a substrate for arrhythmia. This mechanism is studied extensively by B. G. de Barros et al. who present results obtained using two different models: one is heterogeneous with subcellular discretization and the other is a discrete model. Both noncontinuum models show the initiation of reentry inside fibrotic tissue through generation of an ectopic pacemaker using a mixture of excitable and nonexcitable regions. The resulting fractionation of propagation combined with the topology of the mixed region allows an electric wave to reexcite the fibrotic tissue before leaving it. These results may explain previous work suggesting a connection between ectopic pacemakers (or arrhythmia triggers) and complex fractionated electrograms.

Another topic addressed in this special issue is improving the computational performance of algorithms and numerical methods, which is key for simulations of cardiac function that involve large, complex, multiscale, and multiphysics phenomena such as those described above. The use of finite elements for the integration of the continuous cable equations has become very popular because of the freedom it gives in defining the integration domain and the ability to simulate wedges of tissue or the whole heart. G. Cuccuru et al. go a step farther by considering all-hexahedra spectral elements, which result in a more efficient method for 3D monodomain and bidomain representations. Modeling the structure of individual cells and the effects on propagation through the tissue demands efficient microscopic and discrete model representations, as considered in the papers by P. Brocklehurst et al. and B. G. de Barros et al. Adaptation of the spatial and temporal discretization can also increase computational efficiency, as discussed by W. Ying and C. S. Henriquez.

In summary, this special issue addresses some of the most challenging areas of research currently associated with cardiac modeling. We are confident that researchers will find this collection of papers interesting and useful.

*Rodrigo  Weber dos Santos* *Sergio  Alonso* *Elizabeth  M. Cherry* *Joakim  Sundnes*

